# Comparison and Validation of Deep Learning Models for the Diagnosis of Pneumonia

**DOI:** 10.1155/2020/8876798

**Published:** 2020-09-18

**Authors:** Zhenjia Yue, Liangping Ma, Runfeng Zhang

**Affiliations:** ^1^International Education College, Zhengzhou University of Light Industry, Zhengzhou 450000, Henan, China; ^2^School of Mechanical Engineering, Tianjin University, Tianjin 300350, China

## Abstract

As a respiratory infection, pneumonia has gained great attention from countries all over the world for its strong spreading and relatively high mortality. For pneumonia, early detection and treatment will reduce its mortality rate significantly. Currently, X-ray diagnosis is recognized as a relatively effective method. The visual analysis of a patient's X-ray chest radiograph by an experienced doctor takes about 5 to 15 minutes. When cases are concentrated, this will undoubtedly put tremendous pressure on the doctor's clinical diagnosis. Therefore, relying on the naked eye of the imaging doctor has very low efficiency. Hence, the use of artificial intelligence for clinical image diagnosis of pneumonia is a necessary thing. In addition, artificial intelligence recognition is very fast, and the convolutional neural networks (CNNs) have achieved better performance than human beings in terms of image identification. Therefore, we used the dataset which has chest X-ray images for classification made available by Kaggle with a total of 5216 train and 624 test images, with 2 classes as normal and pneumonia. We performed studies using five mainstream network algorithms to classify these diseases in the dataset and compared the results, from which we improved MobileNet's network structure and achieved a higher accuracy rate than other methods. Furthermore, the improved MobileNet's network could also extend to other areas for application.

## 1. Introduction

Pneumonia is an acute respiratory infection of the lungs, and it has a high incidence, accounting for about 12% of the total population. Nowadays, the incidence of pneumonia is still increasing due to the social population aging, increased immune-impaired hosts, pathogen changes, difficult pathogenic diagnosis, and increased bacterial resistance. A chest radiograph analysis (CXR) is the most commonly used method for X-ray examination to diagnose and differentiate the type of pneumonia. However, because of a lack of professional radiologists, pneumonia has alarming mortality rates in some limited resource areas. Therefore, addressing the issue of how to improve the accuracy of pneumonia detection and reducing the cost of pneumonia detection has great help for the treatment and prevention of pneumonia.

In recent years, deep learning technologies have developed rapidly. Deep learning is a widely used tool in research fields such as computer vision, speech analysis, and natural language processing. This method is particularly suitable for those fields that need to analyze large amounts of data and human intelligence. A major advantage of using deep learning methods is that complex features can be learned directly from the raw data. This allows us to define a system that does not rely on manual operations, which is unique among other machine learning technologies. The use of deep learning as a machine learning and pattern recognition tool is also becoming an important aspect in the field of medical image analysis. At present, the deep learning technology has played an important role in medical image processing, computer-aided diagnosis, image interpretation, image fusion, image registration, image segmentation, and image-guided therapy. It can help doctors diagnose and predict disease risk accurately and quickly.

Cicero et al. from St Michael's Hospital discussed the training and validation of CNNs with modest-sized medical data to detect pathology in 2017 [1]. Ma et al. presented a survey on deep learning for pulmonary medical imaging in 2019 [2]. Jaiswal et al. from University of Bedfordshire described an approach based on deep learning for identifying pneumonia in chest X-way in 2019 [3]. Hwang et al. from Seoul National University College of Medicine developed the deep learning-based algorithm for major thoracic diseases on chest radiographs with consistently high performance in 2019 [4]. Sirazitdinov et al. from Innopolis University proposed an ensemble of two convolutional neural networks, namely, RetinaNet and Mask R-CNN, for pneumonia detection and localization, and it is validated to be a liable solution for automated pneumonia diagnosis in 2019 [5]. Rajpurkar et al. from Stanford University developed and validated a deep learning algorithm that classified clinically important abnormalities in chest radiographs at a similar performance level to practicing radiologists in 2018 [6]. Christe et al. from Bern University Hospital proposed the computed-aided detection algorithm based on machine learning which was able to classify idiopathic pulmonary fibrosis as well as man reader in 2019 [7]. Correa et al. from Tulane University School of Public Health and Tropical Medicine presented a method for automatic classification of pneumonia using ultrasound imaging of the lungs and pattern recognition in 2018 [8]. Knok et al. from Polytechnic of Međimurje used an already defined convolution neural network architecture to develop a model of an intelligent system that receives X-ray image of the lung as an input parameter and based on the processed image returned the possibility of pneumonia as an output in 2019 [9]. Rajaraman et al. proposed a CNN-based decision support system to detect pneumonia in pediatric CXRs, and it effectively learned from a sparse collection of complex data with reduced bias and improved generalization in 2018 [10]. Anwar et al. from University of Engineering and Technology, Taxila, reviewed the medical image analysis using convolutional neural networks in 2019 [11]. Professor Razzak et al. from King Saud bin Abdulaziz University for Health Sciences discussed the overview, challenges, and the future of the deep learning for medical image processing in 2018 [12]. Maruyama et al. from Gunma Prefectural College of Health Sciences used three types of machine learning methods to compare their accuracy of medical image classification; their conclusions showed the CNN is more accurate than conventional machine learning methods that utilize the manual feature extraction in 2018 [13]. Gabruseva et al. presented an algorithm that automatically locates lung opacities on chest radiographs by using squeeze-and-excitation CNNs, augmentations, and multitask learning; it demonstrated one of the best performances in the Radiological Society of North America (RSNA) Pneumonia Detection Challenge for pneumonia region detection hosted on the Kaggle platform [14].

Although some of the aforementioned studies use transfer learning methods to solve the limitations of insufficient training data, they have achieved better recognition results in pneumonia image recognition than other studies. However, because of the large difference between the ImageNet dataset and the pneumonia dataset, they did not make corresponding improvements to the existing migration learning model to make it more suitable for the pneumonia image dataset in order to obtain higher recognition accuracy. In addition, all children with pneumonia in the dataset of the previous study are patients with lobar pneumonia, which means that the performance of the algorithm may be affected. In this case, the expected sensitivity is low. In addition, there is currently no algorithm that can determine other types of lung diseases, such as an algorithm that distinguishes interstitial infiltration or bronchiolitis from lobar pneumonia.

In our study, we analyzed the structural advantages of different deep learning models and concluded that MobileNet is a suitable model for clinical image diagnosis of pneumonia. Also, we used the improved MobileNet's network structure for higher accuracy. To validate the theoretical results, we utilize a regular convolution and four other mainstream network models to classify and identify the same pneumonia X-ray datasets acquired in reality. After comparing their accuracy and other performance indicators, the results turn out that improved MobileNet does get better results than other CNNs. In the end, the conclusion and future work are illustrated based on our study.

## 2. Methodology

### 2.1. Depthwise Separable Convolution

**Figure 1:**
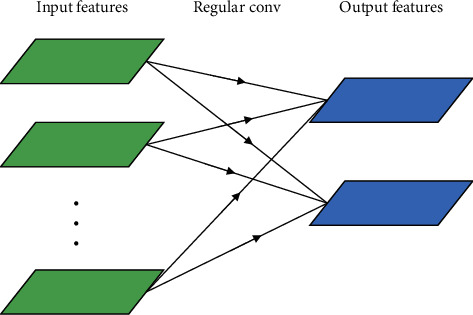
A regular convolution.

A regular convolution is performed in one step by filtering and merging inputs into a new set of outputs (in Figure 1). The depthwise separable convolution divides it into two layers: one layer for filtering and the other layer for merging. The influence of this factorization is to reduce the amount of computation and model size greatly [15].

**Figure 2:**
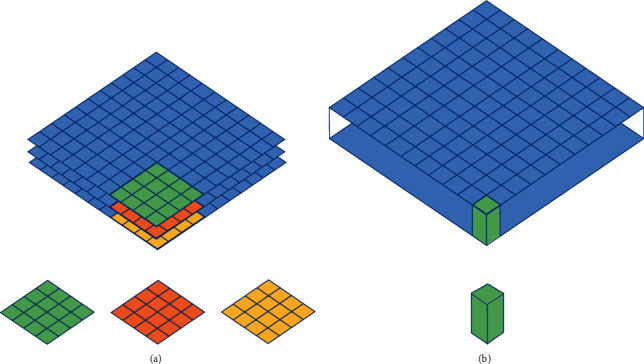
(a) 3D model representation of a depthwise convolution; (b) 3D model representation of a pointwise convolution.

**Figure 3:**
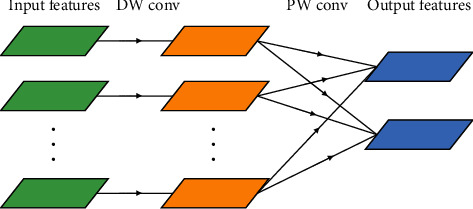
A depthwise separable convolution.

For the depthwise separable convolution, the input images have three channels: red, green, and blue. After several convolutions, the images may have multiple channels. Each channel imaging can be a specific interpretation of the image. For example, the “red” channel explains “red” for each pixel, the “blue” channel explains “blue” for each pixel, and the “green” channel explains “green” for each pixel. An image with 64 channels has 64 different interpretations of the image. Being a distinct regular convolution, a depthwise separable convolution comprises a depthwise convolution (DW) and a pointwise convolution (PW). There, DW deals with spatial relationship modeling with 2D channelwise convolutions (in Figure 2(a)), while PW deals with cross-channel relationship modeling with 1 × 1 convolution across channels (in Figure 2(b)). This factorization form is expressed by DW + PW (in Figure 3).

Next, a depthwise separable convolution will be proved to have better performance by comparing the computational cost of a depthwise separable convolution with a regular convolution.

**Figure 4:**
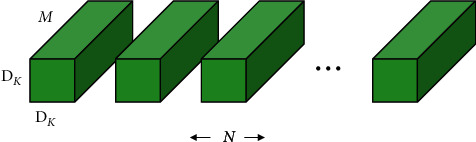
The regular convolution layer.

First, the *D*_*F*_ × *D*_*F*_ × *M* feature map **F** of the regular convolution layer is taken to input, and then a *D*_*F*_ × *D*_*F*_ × *N* feature map **G** (in Figure 4) is generated. *D*_*F*_ represents the spatial width and height of the square input feature map 1, *M* represents the number of input channels (input depth), *D*_*G*_ represents the spatial width and height of the square output feature map, and *N* represents the number of output channels (output depth).

The regular convolutional layer is parameterized by convolution kernel **K** of size *D*_*K*_ × *D*_*K*_ × *M* × *N*. *D*_*K*_ represents the spatial dimension of the kernel assuming a square, *M* represents the number of input channels, and *N* represents the number of output channels.

The output feature map for regular convolution assuming stride 1 and padding is computed as

(1) $$\begin{array}{c} G_{k,l,n}=\sum_{i,j,m}K_{i,j,m,n}\cdot F_{k+i-1,l+j-1,m}. \end{array}$$

The computational cost of the regular convolution **C**_**R**_ is

(2) $$\begin{array}{c} C_{R}=D_{K}\cdot D_{K}\cdot M\cdot N\cdot D_{F}\cdot D_{F}, \end{array}$$

where the computational cost is determined by the number of input channels *M*, the number of output channels *N*, the kernel size *D*_*F*_ × *D*_*F*_, and the feature map size *D*_*F*_ × *D*_*F*_.

**Figure 5:**
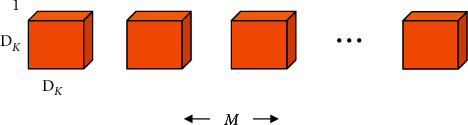
The depthwise convolution layer.

**Figure 6:**
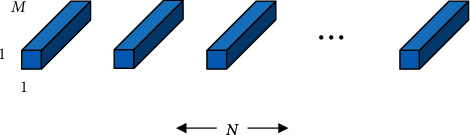
The pointwise convolution layer.

Before, it is introduced that a depthwise separable convolution comprised two layers: a depthwise convolution (in Figure 5) and a pointwise convolution (in Figure 6). The depthwise convolution is used to apply a single filter per each input channel (input depth). Then, we use pointwise convolution, a simple 1 × 1 convolution, to create a linear combination of the output of the depthwise layer. Therefore, we can define the depthwise convolution with one filter per input channel (input depth) as

(3) $$\begin{array}{c} {\hat{G}}_{k,l,m}=\sum_{i,j}{\hat{K}}_{i,j,m}\cdot F_{k+i-1,l+j-1,m}, \end{array}$$

where $\hat{K}$ is the depthwise convolutional kernel of size *D*_*F*_ × *D*_*F*_ × *M*; the *m*_*th*_ filter in $\hat{K}$ is applied to the *m*_*th*_ channel in **F** to produce the *m*_*th*_ channel of the filtered output feature map $\hat{G}$.

The computational cost of the depthwise convolution **C**_**D**_ is

(4) $$\begin{array}{c} C_{D}=D_{K}\cdot D_{K}\cdot M\cdot D_{F}\cdot D_{F}. \end{array}$$

The computational cost of the pointwise convolution **C**_**P**_ is

(5) $$\begin{array}{c} C_{P}=M\cdot N\cdot D_{F}\cdot D_{F}. \end{array}$$

So, the computational cost of the depthwise separable convolution **C**_**DP**_ is

(6) $$\begin{array}{c} C_{DP}=D_{K}\cdot D_{K}\cdot M\cdot D_{F}\cdot D_{F}+M\cdot N\cdot D_{F}\cdot D_{F}, \end{array}$$

which is the sum of the depthwise and 1 × 1 pointwise convolution. By splitting convolution into a 2-step process of filtering and merging, we obtain a reduction **R** in computation of

(7) $$\begin{array}{c} R=\frac{D_{K}\cdot D_{K}\cdot M\cdot D_{F}\cdot D_{F}+M\cdot N\cdot D_{F}\cdot D_{F}}{D_{K}\cdot D_{K}\cdot M\cdot N\cdot D_{F}\cdot D_{F}}=\frac{1}{N}+\frac{1}{D_{K}^{2}}. \end{array}$$

Therefore, it is concluded that the depthwise separable convolution can greatly reduce the amount of computational cost [16].

Moreover, we experimented with reducing the number of filters to reduce redundancy. Howard's network model using 32 filters in a full 3 × 3 convolution is used to build initial filter banks for edge detection. Through the analysis of the experiment results, we found that reducing the number of filters to 16 could maintain the same accuracy as 32 filters, which saves an additional 2 ms.

### 2.2. Model Evaluation Metrics

In order to evaluate the performance of the deep learning model, we refer to the confusion matrix (in Table 1), which is a standard format for expressing accuracy evaluation. Based on this confusion matrix, evaluation is performed using the following criteria:

1. Accuracy represents the ratio of the number of samples correctly classified by the classification model to the total number of samples for a given test data set. It can be expressed by the following formula:

(8) $$\begin{array}{c} \text{Accuracy}=\frac{\text{TP}+\text{TN}}{\text{TP}+\text{TN}+\text{FP}+\text{FN}}. \end{array}$$

(2) Recall represents the positive sample of the original sample, the probability that the classification model correctly predicted a positive sample. It can be expressed by the following formula:

(9) $$\begin{array}{c} \text{Recall}=\frac{\text{TP}}{\text{TP}+\text{FN}}. \end{array}$$

## 3. Experiments

### 3.1. Dataset and Training

**Figure 7:**
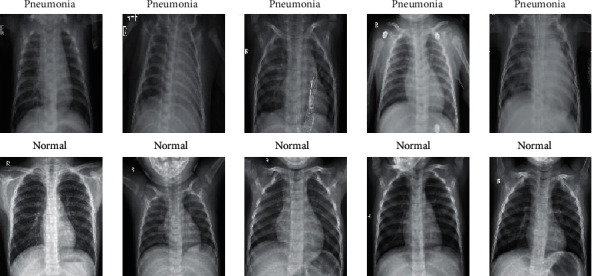
Chest X-ray images (pneumonia/normal).

In this paper, we use the MobileNet which applies 3 × 3 depthwise separable convolutions, a regular CNN, ResNet-18, and two mainstream CNN models pretrained on ImageNet [17]. They are ResNet-50 and VGG19. The dataset was chest X-ray images for classification made available by Kaggle with a total of 5216 train and 624 test images (in Figure 7). The dataset is organized into 3 folders (train, test, and file type) and contains subfolders for each image category (pneumonia/normal). There are 5840 X-ray images (JPEG) and 2 categories (pneumonia/normal).

The chest X-ray images were selected from pediatric patients of one to five years old from Guangzhou Women and Children's Medical Center, Guangzhou. The characteristics of the data and their distribution could be organized (see Table 2). All chest X-rays were performed as part of patients' routine clinical care. This dataset is quality controlled by screening chest X-rays to remove unreadable and low-quality X-rays and is managed by several experts to avoid grading errors.

**Figure 8:**
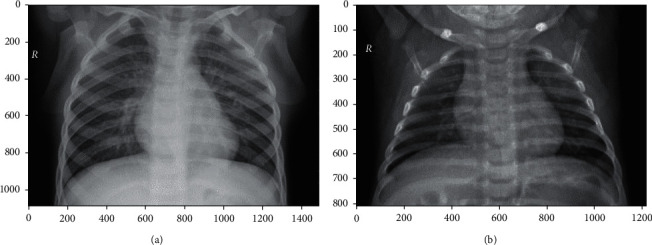
(a) Conversion of the lung with pneumonia image obtained from the dataset into a NumPy array; (b) conversion of the normal lung image obtained from the train set into a NumPy array.

**Figure 9:**
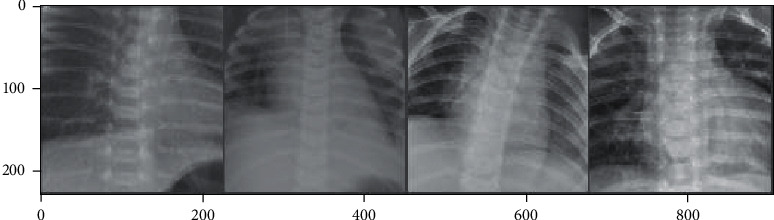
The sizes of images are changed to 226 × 226.

Above all, we should carry out some data analysis and preprocessing. So, we convert the images gotten from the dataset into a NumPy array (see Figure 8). Then, we change the sizes of the images to 226 × 226 in order that we can have more data (images) to train on (in Figure 9). In addition, in order to facilitate comparison, we uniformly set the number of epochs to 20. The experimental environment is an Ubuntu Linux server with GeForce GTX 1050 Ti GPU, and all models are implemented using Python.

## 4. Results

**Figure 10:**
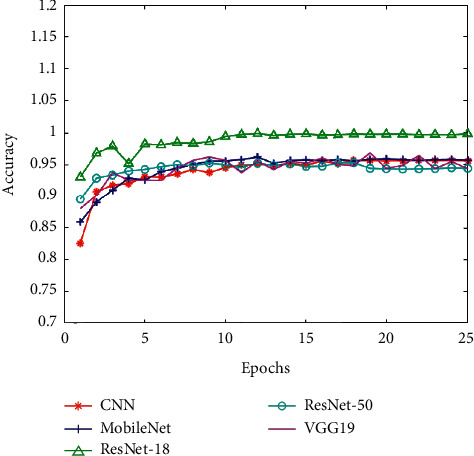
The training set accuracy of five CNNs.

**Figure 11:**
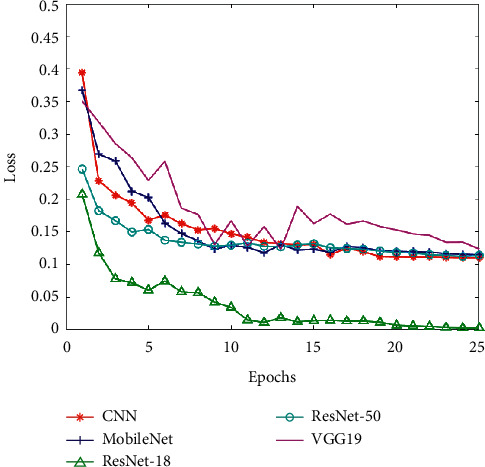
The training set loss of five CNNs.

**Figure 12:**
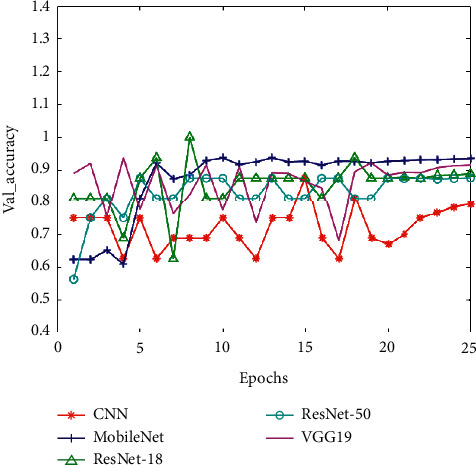
The validation set accuracy of five CNNs.

**Figure 13:**
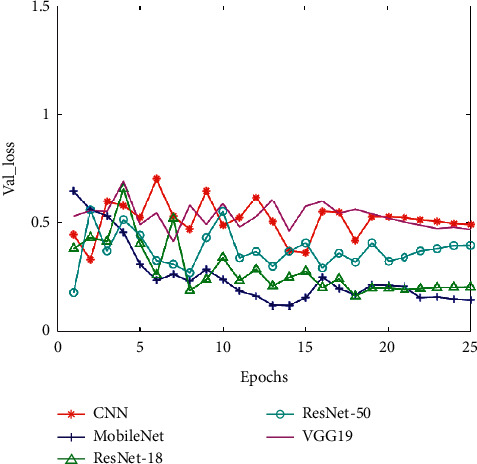
The validation set loss of five CNNs.

Corresponding to these five CNN models, we put the training set accuracy, the training set loss, the validation set accuracy (Val_accuracy), and the validation set loss (Val_loss) in four line charts for comparison (as shown in Figures 10–13). And then, we calculated the average of training set accuracy, training set loss, Val_accuracy, and Val_loss. The results are shown in Table 3. Here, the purpose of setting epoch to 25 is to compare the accuracy of different algorithms under the same number of iterations. This can reflect the speed of training between algorithms. If you encounter a new type of pneumonia and the time is urgent, researchers need to train the lung images of the new type of pneumonia in time, so achieving a higher accuracy rate while saving time is also a factor we need to consider.

After that, we applied the five trained models to the test set for experiments and recorded their accuracy and recall. The result indicates that the accuracy of pneumonia recognition using MobileNet is up to 92.79% and the recall is 98.90% (see Table 4). In addition, we can see from Figures 10–13 that the MobileNet has higher accuracy and lower loss and at the same time has less floating-point calculations. Compared with other networks, MobileNet can trade for better data throughput while sacrificing very little accuracy.

## 5. Discussion

From the experimental results, it can be seen that MobileNet, as a lightweight network, not only has a smaller amount of calculations than most CNNs but also has a better classification effect than other types of CNN models when the number of parameters is almost on an order of magnitude. This benefits from using the depthwise separable convolution. Since the development of deep learning, most image recognition models have large parameters and a large amount of calculations, which are not suitable for use in embedded devices. For the identification of pneumonia, a common disease, we must also consider how to quickly and accurately identify pneumonia in areas where equipment and doctors are scarce. This is one of the reasons why we recommend using MobileNet for pneumonia recognition.

## 6. Conclusions

In this paper, five mainstream deep learning models are used to diagnose clinical data on a dataset consisting of X-ray images of the lungs with pneumonia and normal lungs and the accuracy of these methods is compared. Among them, because of the superior performance of MobileNet, we focus on the network structure of MobileNet. The results demonstrated that all five network structures have the ability to recognize pneumonia and the accuracy of MobileNet is higher than other network structures. In addition, the application of artificial intelligence technology in the medical field is not sufficient, and the dataset in this field should be improved in terms of types. As the amount of pneumonia image data increases and the network structure continues to improve, the performance of CNN-based pneumonia diagnosis algorithms will also continue to improve. In the future, the application of clinical image diagnosis of pneumonia X-rays can reduce the workload of clinicians and enable patients to obtain early diagnosis and timely treatment, thereby reducing the mortality rate of pneumonia.

## Figures and Tables

**Table 1:** Confusion matrix.

	Actual positive	Actual negative
Predicted positive	TP	FP
Predicted negative	FN	TN

**Table 2:** The data characteristics of the dataset.

Category	Train samples	Test samples	File type
Normal	1341	234	JPEG
Pneumonia	3875	390	JPEG

**Table 3:** The five CNNs' average of their training set accuracy, training set loss, validation set accuracy, and validation set loss.

	Accuracy	Loss	Val_accuracy	Val_loss
MobileNet	0.94454	0.15300	0.87119	0.25509
ResNet-18	0.98795	0.03783	0.85388	0.28453
ResNet-50	0.94342	0.13564	0.82982	0.37387
VGG19	0.94318	0.18500	0.86044	0.50610
CNN	0.93980	0.15152	0.72090	0.51290

**Table 4:** The five CNNs' testing accuracy and recall.

	Accuracy	Recall
MobileNet	0.92986	0.98984
ResNet-18	0.85515	0.98947
ResNet-50	0.87486	0.98531
VGG19	0.90529	0.78635
CNN	0.91446	0.98813

## Data Availability

The dataset used in this study was chest X-ray images by Kaggle, please visit https://www.kaggle.com/paultimothymooney/chest-xray-pneumonia.
